# Increased Risk for Developing Major Adverse Cardiovascular Events in Stented Chinese Patients Treated with Dual Antiplatelet Therapy after Concomitant Use of the Proton Pump Inhibitor

**DOI:** 10.1371/journal.pone.0084985

**Published:** 2014-01-08

**Authors:** Jian-Jun Zou, Shao-Liang Chen, Jie Tan, Ling Lin, Ying-Ying Zhao, Hai-Mei Xu, Song Lin, Juan Zhang, Hong-Wei Fan, Hong-Guang Xie

**Affiliations:** 1 Division of Clinical Pharmacology, General Clinical Research Center, Nanjing Medical University Nanjing First Hospital, Nanjing, China; 2 Division of Cardiovascular Medicine, Department of Medicine, Nanjing Medical University Nanjing First Hospital, Nanjing, China; 3 Central Laboratory, General Clinical Research Center, Nanjing Medical University Nanjing First Hospital, Nanjing, China; 4 Department of Pharmacology, Nanjing Medical University School of Pharmacy, Nanjing, China; University of Buenos Aires, Faculty of Medicine. Cardiovascular Pathophysiology Institute., Argentina

## Abstract

**Background:**

Some clinical studies have demonstrated that the proton pump inhibitor (PPI) could decrease clopidogrel platelet response and increase major adverse cardiovascular events (MACE) in white or black subjects. However, that remains to be determined in Chinese patients. In this study, we sought to determine whether there could be an increased risk for developing MACE after concomitant use of dual antiplatelet therapy (DAT) and a PPI in Chinese patients treated with percutaneous coronary intervention (PCI) and stenting.

**Methods:**

This study was a 5-year, single-center, retrospective cohort analysis of eligible patients (n = 6188) who received DAT and a PPI concomitantly (defined as PPI users) before discharge and/or 12-month follow-up after discharge as compared with those who received DAT alone (also defined as non-PPI users, n = 1465). The incidence of recurrent MACE, such as myocardial infarction (MI), definite stent thromboses (ST), or cardiovascular death, was compared between the PPI users and non-users.

**Results:**

PPI users had a significantly higher incidence of the MACE than non-users (13.9% vs. 10.6%; adjusted HR: 1.33; 95% CI: 1.12 – 1.57, P = 0.007). Stratified analysis revealed that concurrent use of DAT and a PPI was associated with a significantly increased risk for developing ST compared with DAT alone (1% vs. 0.4%; adjusted HR: 2.66, 95% CI: 1.16 – 5.87, P = 0.012). However, there were no significant differences in the risk of MI, cardiovascular death and other adverse events, regardless of combination of clopidogrel and a PPI.

**Conclusions:**

The study further suggests that concomitant use of DAT and a PPI may be associated with an increased risk for developing MACE, in particular definite ST, in Chinese PCI patients after discharge as compared with use of DAT alone.

## Introduction

Clopidogrel, an oral antiplatelet agent, is extensively used to prevent adverse cardiovascular events in patients with acute coronary syndromes (ACS) or those undergoing percutaneous coronary intervention (PCI) for stenting [1]. As a prodrug, clopidogrel needs biotransformation into its active metabolite in the liver [2]. After metabolic bioactivation, clopidogrel active metabolite in plasma irreversibly binds to platelet ADP receptor P2Y12, and consequently suppresses ADP-induced platelet aggregation. This conversion is catalyzed by several cytochrome P450 (CYP) enzymes, of which CYP2C19 is the most important [3]. When taking clopidogrel, carriers of the loss-of-function polymorphisms in the *CYP2C19* gene would have less formation of clopidogrel active metabolite, attenuated platelet inhibition, and increased risk of adverse cardiovascular events as compared with non-carriers [4]–[5].

The proton pump inhibitor (also known as PPI) is often concomitantly prescribed for patients who are being treated with dual antiplatelet therapy (DAT, clopidogrel and aspirin) to reduce the risk of upper gastrointestinal (GI) bleeding complications [6]–[7]. PPIs and clopidogrel share common metabolic pathways mediated by CYP2C19 and CYP3A4 [8]–[9], and therefore, concurrent use of the PPI and clopidogrel can competitively inhibit the conversion of clopidogrel to its active metabolite, leading to reduced platelet inhibition. Several pharmacodynamic studies have shown that some PPIs, in particular omeprazole, could decrease clopidogrel platelet response [10]–[12]. That can be explained by the fact that PPIs can inhibit CYP2C19 activity [8], and that CYP2C19 plays an important role in the CYP2C19-mediated activation of clopidogrel [10]–[12].

However, it remains inconclusive that there is an increased risk of major adverse cardiovascular events (MACE) in PCI patients treated with DAT when taking a PPI concomitantly. For example, several clinical studies have demonstrated that PPI users are often (but not always) associated with an increased risk of MACE in DAT-treated patients with coronary artery disease (CAD) as compared with non-PPI users [13]–[19]. In view of that inconsistency, the US FDA highlights the need for additional studies to evaluate clinical efficacy of clopidogrel when used concomitantly with a PPI. In addition, Chinese populations have a significantly higher frequency of the *CYP2C19* loss-of-function variant alleles (*CYP2C19* *2 and *3) than white subjects [20]–[21], it could be deduced that clopidogrel-treated Chinese patients are more likely to have amplified inhibition of clopidogrel active metabolite formation and attenuated suppression of ADP-induced platelet aggregation as compared with white patients when taking a PPI concomitantly to prevent DAT-associated bleeding; however, clinical relevance of these two drugs to the observed increased risk of MACE in PCI patients of Chinese descent remains to be determined. To further bridge this gap, we compared 1-year adverse clinical outcomes between patients taking clopidogrel alone or in combination with a PPI before discharge and/or after discharge in a cohort of Chinese patients.

## Methods

### Study populations and protocol

**Figure 1:**
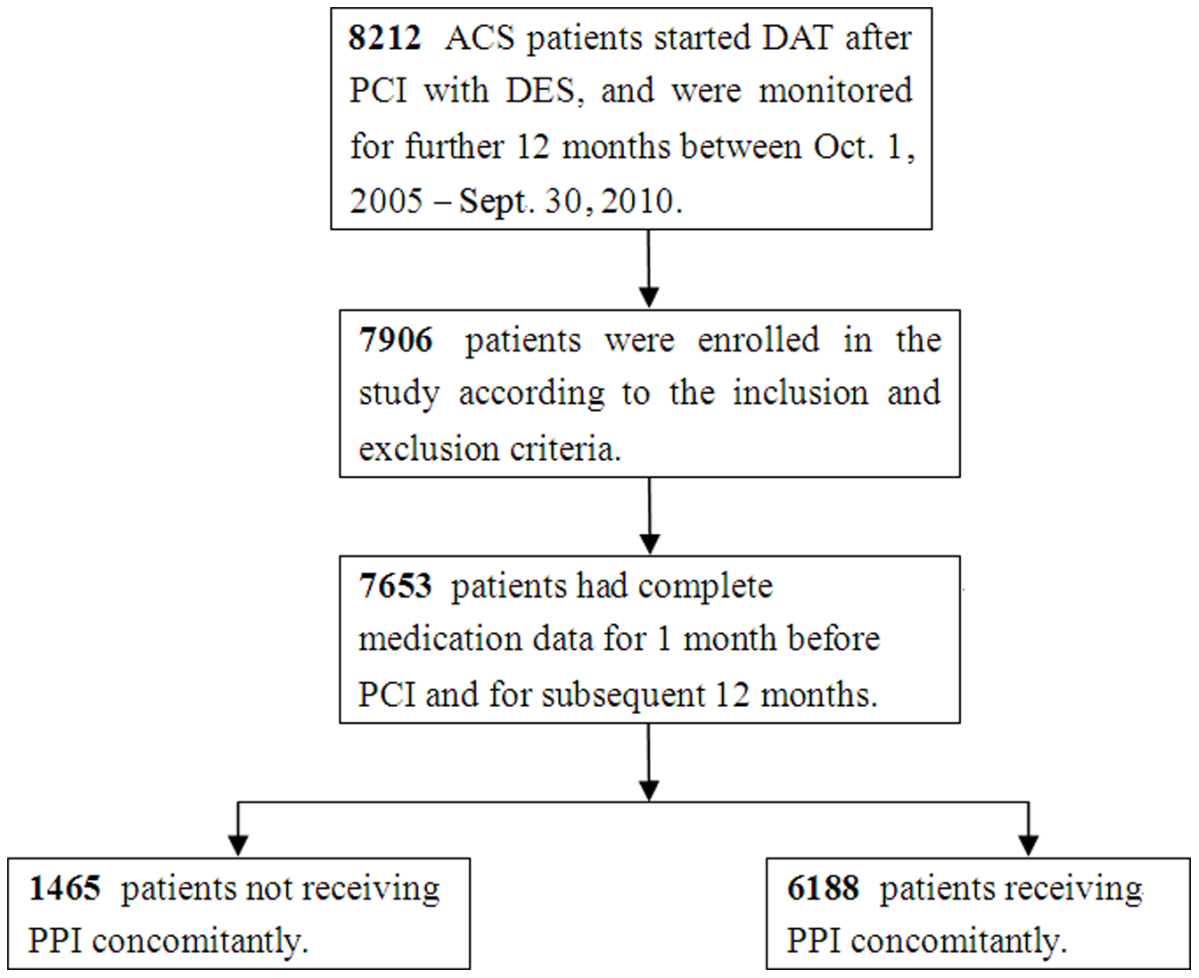
Schematic of the patient selection procedure. ACS, acute coronary syndromes; PCI, percutaneous coronary intervention; DES, drug-eluting stent; PPI, proton pump inhibitor.

Co-medication of the DAT and a PPI was used to prevent DAT-associated bleeding complications in patients who had a history of prior GI ulcer and bleeding symptoms, or increased susceptibility to bleeding. This work was a post hoc analysis of a 5-year, single-center, retrospective, observational cohort study of 8,212 Han Chinese patients treated with drug-eluting stent (DES) placement. The clinical observational period was October 1, 2005 to September 30, 2010. Consecutive clopidogrel on-treatment patients (aged 18 – 75 years) were eligible for the further evaluation of a cohort of patients enrolled in the Division of Cardiovascular Medicine, Department of Medicine, Nanjing First Hospital, Nanjing Medical University, China. To be included in this clinical research study, patients had to have complete medication data for 1 month before PCI and stent, and for 12-month follow-up thereafter. After hospital discharge, patients would be excluded if they had interrupted clopidogrel medication or were not on clopidogrel. In other words, each enrolled patient should highly adhere to receiving clopidogrel for entire 12 months before and after discharge, and patients with no discharge medication data were excluded from the analysis. For this reason, all participants were given orally with a loading dose of 300 mg aspirin and 300 mg clopidogrel before PCI and stenting and then received 12-month DAT with daily maintenance doses of aspirin (100 mg, for long-life) and clopidogrel (75 mg, for 12 months) after discharge. Further inclusion criteria of the PPI users were that they received at least 3 prescriptions of a PPI, or took a PPI of more than 6 days, regardless of before and at discharge, throughout the 12-month follow-up period, or both. A PCI patient who never took a PPI or did not meet the above inclusion criteria was categorized as a non-PPI user. Using the prescription records, including hospitalized medical records at discharge, outpatient clinical visits, questionnaires or telephone interview during the follow-up period, we systematically evaluated exposure of each patient to clopidogrel, aspirin, and/or a PPI within 1 year after discharge. Patients were grouped into PPI users (n = 6188) or non-users (n = 1465) as summarized in **Fig. 1**. A total of 6,188 PPI users were further sub-classified as follows: 1) 6052 patients (97.8%) took a PPI before discharge after PCI and throughout the whole period of 12-month follow-up; 2) 136 patients (2.2%) took a PPI just throughout the follow-up period, but not before and at discharge; and 3) there were no patients who took a PPI only before and at discharge, rather than in the whole follow-up period.

Uninterrupted medication of clopidogrel and aspirin was confirmed at assigned time points of the follow-up period. Patients who discontinued use of clopidogrel for any reasons other than the occurrence of MACE or death were excluded in advance from this study. In addition, more exclusion criteria were as follows: active bleeding, platelet count <100 ×10^9^/L, severe hepatic or renal disorders, prior coronary artery bypass graft (CABG), active malignancy, body mass index (BMI) <18.5 or >40 kg/m^2^, contraindications to use of aspirin or clopidogrel, and premature clopidogrel or aspirin cessation.

The study protocol and subsequent data collection were approved by the ethics committee of Nanjing First Hospital, Nanjing Medical University, China, and all patients signed their written informed consent for cardiovascular intervention before participation.

### Data Collection of Clinical Research Study

The records of hospitalized patients included detailed information on the dates of hospital admission and discharge, discharge diagnosis, specified treatment procedures, co-medication, and clinical efficacy before discharge and 12-month follow-up after discharge. For enrolled patients, we systematically evaluated their demographic characteristics and baseline data, including age, gender, BMI, lifestyle habits, biochemical testing, potential risk factors (e.g., hypertension, diabetes mellitus, dyslipidemia, and cigarette smoking) and concurrent medications at discharge. In addition, medication before and after discharge, in particular the PPI and clopidogrel, was reviewed for this analysis. All information from their attending physicians, relatives, and hospital managers was retrieved.

Collected patient data were input into the electronic database by well-trained staff unaware of the study protocol and grouping. Data quality was randomly monitored to determine whether there were any inconsistency and errors between electronic database and actual data sheets, and subsequent data audit was performed at a regular interval.

After discharge, angiography was routinely scheduled at 6 and 12 months, or earlier than scheduled if needed. Clinical follow-up data was obtained using prescription records, reviewing of hospital medical records, outpatient clinical visits, written questionnaires, and telephone interview during 12 months after stent placement, respectively.

### Primary and Secondary Endpoints

We measured the occurrence of clinical endpoints before discharge and over up to 12-month follow-up: MACE, nonfatal myocardial infarction (MI), definite stent thrombosis (ST), target vessel revascularization (TVR), target lesion revascularization (TLR), CABG, and all-cause death. Primary endpoint was the occurrence of MACE, defined as a composite of death, MI, TVR, TLR, CABG, or ST over 12 months after stenting. All death cases were considered cardiac unless otherwise documented. MI was diagnosed if increased plasma CK-MB levels doubled its baseline value immediately before stenting in acute MI patients or new abnormal Q-wave was detected with ECG. TVR, TLR, and CABG were defined according to the Academic Research Consortium (ARC) definitions [22], and Double Kissing Crush criteria [23], respectively. ST was defined as the occurrence of ACS with angiographic confirmation of thrombosis according to the ARC criteria [24]. In contrast, a secondary endpoint was defined as a single component of the MACE, in particular definite ST. These clinical endpoints were analyzed and adjudicated by the members of an independent committee blinded to the study protocol and grouping.

### Statistical Analysis

Data are expressed as mean ± SD or number (percentage). Categorical values were analyzed with Chi-square test. Continuous variables with a Gaussian distribution were compared by means of the unpaired two-tailed Student's *t* test, whereas continuous variables with a non-Gaussian distribution were compared by Mann-Whitney U test. Multivariate logistic regression analysis was used to compare primary and secondary endpoints between groups with and without PPI. Factors that were identified through univariate analysis (P <0.20) and other potential confounding factors that were considered likely to have an important prognostic value were tested by multivariate logistic regression for association with clinical adverse cardiovascular events during the follow-up period. All statistical analyses were performed with SPSS 16.0 (SPSS Inc., Chicago, IL, USA). Statistical significance was accepted at two-tailed P<0.05.

## Results

### Baseline Characteristics of Enrolled Patients

As summarized in **Fig. 1**, a total of 7,906 patients were chosen from the electronic database for further analysis according to prespecified inclusion and exclusion criteria. Of them, 7653 (96.8%) had complete information on continuous medications before and after discharge and thus were included in the analysis, whereas 253 patients (3.2%) were excluded. All patients were hospitalized for PCI and stenting for 5.9 ± 3.1 days, with a range of 2 – 18 days and a median of 5. According to the prescription records, all PPI users had at least more than three prescriptions of a PPI for 42.6±29.2 days, with a range of 6 – 301 days and a median of 40. Furthermore, all 7653 eligible patients were categorized into two groups: PPI users (n = 6188) and non-PPI users (n = 1465) according to pre-specified definition of the PPI user versus non-user. Of the PPI users, 5587 (90.3%) were on omeprazole at and/or after discharge, 407 (6.6%) on pantoprazole, and 194 (3.1%) on esomeprazole. Summarized in **Table 1** are their baseline values, demographic characteristics, risk factors and concurrent drug therapy. Patients with concomitant PPI use were slightly older (P = 0.094), and had a higher proportion of GI disorder (P = 0.003) but a lower frequency of the presence of prior MI (P = 0.027) or impaired LVEF (P = 0.05) than those without. However, there were no differences in the demographics characteristics, cardiovascular risk factors and discharge medications except for less use of an angiotensin-converting enzyme inhibitor (P = 0.001) and more frequent use of calcium channel blocker (P = 0.022) in the PPI users than non-users.

**Table 1:** Baseline characteristics and clinical profiles of clopidogrel-treated at hospital discharge.

	PPI user (n = 6188)	Non-PPI user (n = 1465)	P value
Age, yrs	66.2±10.2	65.7± 10.6	0.094
Male, n (%)	4548 (73.5)	1083 (73.9)	0.738
BMI, kg/m^2^	25.1±3.2	25.2±3.8	0.300
Current smoking, n (%)	1993 (32.2)	454 (31.0)	0.368
Hypertension, n (%)	4412 (71.3)	1031 (70.4)	0.484
Hyperlipidemia, n (%)	3725 (60.2)	913 (62.3)	0.134
Diabetes mellitus, n (%)	1597 (25.8)	346 (23.6)	0.081
Previous MI, n (%)	1071 (17.3)	290 (19.8)	**0.027**
Stable angina, n (%)	842 (13.6)	189 (12.9)	0.475
Unstable angina, n (%)	4028 (65.1)	990 (67.6)	0.071
GI disease, n (%)	637 (10.3)	114 (7.8)	**0.003**
WBC count, ×10 ^12^ /L	7.8±2.1	7.9±2.2	0.104
Platelet count, ×10 ^9^/L	218.6±60.5	219.1± 61.3	0.777
Creatinine, mg/dL	98±19	99±18	0.067
LDL-C, mmol/L	2.23±0.80	2.26±0.85	0.202
LVEF<40%	1368 (22.1)	359 (24.5)	**0.050**
Using abciximab, n (%)	124 (2.0)	32 (2.2)	0.663
Using a statin, n (%)	5724 (92.5)	1373 (93.7)	0.100
Using an ACEI, n (%)	2364 (38.2)	627 (42.8)	**0.001**
Using a beta-blocker	1046 (16.9)	242 (16.5)	0.723
Using a CCB, n (%)	2426 (39.2)	527 (36.0)	**0.022**
Using a nitrate, n (%)	2704 (43.7)	675 (46.1)	0.100
Total no. stents	2.5±1.8	2.6±1.8	0.056
Diameter of stent, mm	3.02±0.45	3.04±0.43	0.123
Total length of stent, mm	45.0±32.2	45.3±33.1	0.750

Values are mean ± SD or n (%). PPI, proton pump inhibitor; BMI, body mass index; GI, gastro-intestinal; MI, myocardial infarction; LDL-C, low-density lipoprotein-cholesterol; LVEF, left ventricular ejection fraction; ACEI, angiotensin-converting enzyme inhibitor; CCB, calcium channel blocker.

### Clinical Endpoints of Interest

Adverse clinical outcomes of chosen eligible patients were summarized and compared between the two groups. As shown in **Table 2**, PPI users had a higher incidence of adverse cardiovascular events than non-PPI users. For example, the MACE occurred in 13.9% of the PPI users versus in 10.6% of non-users, with an unadjusted hazard ratio (HR) of 1.36 (95% CI: 1.14 – 1.64, P = 0.001). Moreover, multivariable Logistic regression analysis revealed that concomitant use of clopidogrel and a PPI was independently associated with an increased risk for developing MACE (adjusted HR, 1.33; 95% CI: 1.12 – 1.57, P = 0.007) or ST events (adjusted HR: 2.66; 95% CI: 1.16–5.87; P = 0.012). However, concomitant use of clopidogrel and a PPI was not associated with an increased risk for developing MI, cardiovascular death, TVR, TLR, or CABG during the follow-up period, and these outcomes did not change after adjustment by multivariate Logistic regression analysis. PPI users had an increased tendency in the risk for developing MI (1.1% vs. 0.6%, P = 0.068) and TVR (6.9% vs. 5.6%, P = 0.066) as compared with non-users.

**Table 2:** The incidence of MACE in clopidogrel-treated patients enrolled in the study.

Adverse event	PPI user (n = 6188)	Non-PPI user (n = 1465)	Unadjusted HR (95% CI)	P value	Adjusted HR (95% CI)	P value
MACE, n (%)	860 (13.9)	155 (10.6)	1.36 (1.14–1.64)	0.001	1.33 (1.12–1.57)	**0.007**
ST, n (%)	63 (1.0)	6 (0.4)	2.50 (1.08–5.79)	0.015	2.66 (1.16–5.87)	**0.012**
MI, n (%)	69 (1.1)	9 (0.6)	1.82 (0.91–3.66)	0.068	1.79 (0.88–3.72)	0.095
Death, n (%)	223 (3.6)	63 (4.3)	0.83 (0.62–1.11)	0.214	0.92 (0.59–1.23)	0.342
TVR, n (%)	427 (6.9)	82 (5.6)	1.25 (0.98–1.59)	0.066	1.60 (0.45–3.17)	0.129
TLR, n (%)	340 (5.5)	72 (4.9)	1.12 (0.87–1.46)	0.371	0.89 (0.39–2.08)	0.757
CABG, n (%)	37 (0.6)	7 (0.5)	1.25 (0.56–2.82)	0.576	1.36 (0.67–2.91)	0.453

PPI, proton pump inhibitor; HR, hazard ratio; CI, confidence interval; MACE, major adverse cardiovascular event; ST, stent thromboses; MI, myocardial infarction; TVR, target vessel revascularization; TLR, target lesion revascularization; CABG, coronary artery bypass graft.

‘Potential confounding factors’ were age, hyperlipidemia, diabetes mellitus, previous MI, unstable angina, GI disease, creatinine, LVEF, ACEI, CCB and total number of stents.

## Discussion

The PPIs are often prescribed for the prophylaxis of serious upper GI bleeding complications when DAT is used to prevent recurrent MACE in CAD patients. Clinical studies have demonstrated that concomitant use of clopidogrel and a PPI is frequently (but not always) associated with an increased risk for developing MACE in patients of European or black African descent [13], [15]–[19]. In this retrospective cohort analysis of Han Chinese PPI users (n = 6188) versus non-users (n = 1465), we observed that PPI users had a significantly higher incidence of the MACE or ST events than non-users in a Chinese patient population. However, there were no significant differences in the risk of MI, cardiovascular death and other adverse events, regardless of combination of clopidogrel and a PPI, which is consistent with the findings derived from other ethnic populations.

Evidence has documented that CYP2C19 plays an important role in clopidogrel bioactivation in the liver [10]–[12], [20], and that some (if not all) PPIs can inhibit CYP2C19 activity [8] and affect clopidogrel platelet response [10]–[12], [25]-[27]. To further exclude the potential impact of known covariates (such as coexisting diseases, drug interactions, and marked heterogeneity of recruited patients) on the platelet response to clopidogrel, healthy subjects should be chosen to determine such a drug interaction. As expected, there was higher residual platelet aggregation in healthy subjects when they received clopidogrel and omeprazole or lansoprazole concomitantly than clopidogrel alone [10], [28]; however, such an attenuated antiplatelet effect may differ by either individual PPIs or *CYP2C19* genotype [28].

It is generally recognized that a randomized controlled trial (also known as RCT) is the gold standard of clinical research studies. By a RCT approach, Gilard et al. found that omeprazole reduced clopidogrel-induced antiplatelet effect [25]. Contrary to the above, in another randomized, placebo-controlled clinical trial of 3761 CAD patients treated with either DAT plus placebo or DAT plus omeprazole for 180 days, no apparent cardiovascular interaction between clopidogrel and omeprazole was observed after evaluation of the incidence of clinical effects associated with the use of the PPI (the cardiovascular event rate: 5.7% with placebo vs. 4.9% with omeprazole; HR with omeprazole: 0.99; 95% CI: 0.68 to 1.44; P = 0.96) [29]. However, other clinical research studies have provided evidence suggesting that adverse cardiovascular interaction of a PPI with clopidogrel may be translated into worse clinical outcomes [10], [25]–[27].

In a cohort of 6188 Chinese PPI users versus 1465 non-users, we observed that 5587 PPI users (90.3%) were on omeprazole at or after discharge, 407 (6.6%) on pantoprazole, and 194 (3.1%) on esomeprazole, suggesting that omeprazole made the major contribution to the interaction of the PPIs with clopidogrel in Chinese ACS patients who were treated PCI and DES. Moreover, PPI users had a significantly higher incidence of the MACE or ST events than non-users, which is consistent with the findings derived from a retrospective cohort analysis of 8205 black and white ACS patients taking clopidogrel alone or in combination with a PPI after discharge [13]. In another retrospective analysis of 18565 patients receiving clopidogrel after PCI showed an adjusted HR of 1.22 for the primary endpoint of death or MI in patients receiving a PPI and clopidogrel concomitantly for 180 days [19]. These clinical oberservations indicate that after concurrent use of a PPI and clopidogrel, patients may be at increased risk for recurrent adverse cardiovascular events as compared with use of clopidogrel alone, suggesting that concurrent use of the PPI and clopidogrel may be associated with impaired benefits of clopidogrel [13], [28], [30].

Bleeding complication is an extension of the antiplatelet effect of the drug, and thus combination of DAT and a PPI is often recommended to prevent GI bleeding in patient care [20]. It is not surprising that PPI uses had a markedly high frequency of GI disorders (including GI ulcer and/or bleeding) than non-users as shown in **Table 1**. Because PPI users had a less frequency of prior MI and impaired LVEF than non-users before discharge, a significantly higher incidence of MACE or ST events in PPI users than non-users in this “real world” clinical practice may suggest that the presence of a PPI would worsen cardiovascular effects of clopidogrel due to their drug interactions.

This study had some limitations that would be worth further discussion. First of all, lack of exact and complete information on concomitant use of the PPI in clopidogrel-treated patients after discharge was a major limitation in such retrospective clinical research studies, because some patients took the PPI irregularly or even intermittently after discharge, and the PPI exposure status of each patient might be misclassified. Therefore, extreme caution should be taken with the conclusion derived from the results of a retrospective cohort analysis. Second, we could not completely exclude possible selection bias of patients in a retrospective observational clinical study. Although considerable efforts had been made to minimize the influence of known confounders on unknown results, there could be other unknown factors that may affect results to be observed. Also because this study was a post hoc analysis of a clinical cohort, it was subjected to the limitations inherent to all relevant analysis; however, the multivariable adjustment model confirmed the primary analyses. Third, we did not use *CYP2C19* genotype status as a known confounder that could affect clopidogrel platelet response and/or clinical outcomes. Finally, because of incomplete information on PPI prescriptions, we did not do stratified analysis of individual PPIs, although PPIs did not exhibit a class effect on clopidogrel platelet response. However, the major individual of the PPIs used in Chinese patients was omeprazole (90.3%). Therefore, the conclusion cannot be extrapolated to other PPIs.

In conclusion, this retrospective cohort study further suggests that concomitant use of clopidogrel and a PPI (predominantly omeprazole) after PCI may be associated with an increased risk of the MACE, in particular ST, in the 12-month follow-up after discharge in Chinese patients with ACS, and that inhibition of CYP2C19 by some (not all) PPIs may, in turn, result in attenuated clopidogrel platelet response and increased adverse cardiovascular events.
